# ICAT mediates the inhibition of stemness and tumorigenesis in acute myeloid leukemia cells induced by 1,25-(OH)_2_D_3_

**DOI:** 10.32604/or.2024.051746

**Published:** 2025-02-28

**Authors:** YULIAN WANG, LIANLI ZHU, RONGHAO ZENG, YUNPING PU, BAIJIAN CHEN, YUWEI TAN, MING HONG, WEIJIA WANG

**Affiliations:** 1 Graduate School, Zhuhai Campus of Zunyi Medical University, Zhuhai, 519041, China; 2 Department of Advanced Diagnostic and Clinical Medicine, Zhongshan People’s Hospital, Zhongshan, 528403, China; 3 Graduate School, Guangdong Medical University, Zhanjiang, 524023, China

**Keywords:** β-catenin–interacting protein 1 (ICAT), 1,25-dihydroxyvitamin D3 (1,25-(OH)_2_D_3_), Acute myeloid leukemia (AML), Stemness

## Abstract

**Background:**

The role of 1,25-dihydroxyvitamin D3 (1,25-(OH)_2_D_3_) in cancer prevention and treatment is an emerging topic of interest. However, its effects on the stemness of acute myeloid leukemia (AML) cells are poorly understood.

**Methods:**

The proliferation and differentiation of AML cells (HL60 and NB4) were investigated by the CCK-8 assay, immunocytochemical staining, and flow cytometry. The abilities of HL60 and NB4 cells to form spheres were examined by the cell sphere formation assay. In addition, the levels of stemness-associated markers (SOX2, Nanog, OCT4, and c-Myc) in HL60 and NB4 cells were measured by western blotting and quantitative real-time polymerase chain reaction. Moreover, we obtained β-catenin-interacting protein 1 (ICAT)-knockout and ICAT-overexpressing HL-60 cells using gene editing and lentiviral infection techniques and investigated the role of ICAT in modulating the stemness-inhibiting effects of 1,25-(OH)_2_D_3_ using the aforementioned experimental methods. Finally, we validated our findings *in vivo* using NOD/SCID mice.

**Results:**

1,25-(OH)_2_D_3_ inhibited the proliferation and stemness of AML cells (HL60 and NB4) and induced their differentiation into monocytes. Additionally, the knockdown of ICAT in HL60 cells attenuated the inhibitory effects of 1,25-(OH)_2_D_3_ on proliferation and stemness and suppressed the expression of stemness markers. Conversely, overexpression of ICAT enhanced the aforementioned inhibitory effects of 1,25-(OH)_2_D_3_. Consistently, in NOD/SCID mice, 1,25-(OH)_2_D_3_ suppressed tumor formation by HL-60 cells, and the effects of ICAT knockdown or overexpression on 1,25-(OH)_2_D_3_ aligned with the *in vitro* findings.

**Conclusion:**

1,25-(OH)_2_D_3_ inhibits AML cell stemness, possibly through modulation of the ICAT-mediated Wnt/β-catenin signaling pathway.

## Introduction

Acute myeloid leukemia (AML) is a type of blood and bone marrow cancer characterized by the rapid accumulation of abnormal white blood cells, known as myeloblasts. These cells interfere with the production of normal blood cells, leading to symptoms such as fatigue, infections, and easy bruising or bleeding. AML can affect people of all ages but is more common in older adults [1]. AML comprises a complex group of diseases. The WHO has proposed a classification of acute leukemia that incorporates genetic, epidemiological, and immunologic factors in addition to morphological factors. The latest classification also includes molecular analyses [2]. Current therapeutic strategies for AML include chemotherapy, hematopoietic stem cell transplantation, and immunotherapy [3]. However, conventional chemotherapy often fails to achieve long-term remission, and disease relapse is frequent [4]. Studies have illustrated that stemness, the ability of leukemia cells to self-renew and resist differentiation, is a major driver of primary drug resistance in AML [5]. Addressing both stemness and specific high-risk co-mutations could potentially circumvent resistance and enhance survival outcomes for individuals diagnosed with AML [5]. Therefore, targeting the stemness of leukemia cells is particularly crucial in the context of treatment resistance, prognosis, and relapse in AML.

1,25-(OH)_2_D_3_, also known as 1α,25-dihydroxyvitamin D_3_, is a multifunctional hormone and a key controller of human genetic activity. It influences the characteristics and functions of different types of cells by regulating the activity of numerous genes in a specific manner depending on the tissue and cell type [6]. Prior studies indicated that 1,25-(OH)_2_D_3_ can regulate innate and adaptive immunity as well as calcium and bone homeostasis [7–9]. Studies also reported that 1,25-(OH)_2_D_3_ can inhibit the stemness of cancer cells. Specifically, 1,25-(OH)_2_D_3_ has inhibitory effects on tumor cell proliferation and promotive effects on the differentiation of tumor cells, including colorectal cancer, melanoma, and leukemia cells [10–12]. In addition, 1,25-(OH)_2_D_3_ has been demonstrated to attenuate the stemness of tumor cells, including ovarian cancer cells, pancreatic cancer cells, breast cancer cells, and glioblastoma stem-like cells [13–17]. 1,25-(OH)_2_D_3_ exerts anticancer effects by modulating multiple signaling pathways to inhibit tumor cell stemness. Previous research highlighted the pivotal role of the Wnt/β-catenin signaling in mediating the suppressive effects of 1,25-(OH)_2_D_3_ on the stemness of breast cancer and ovarian cancer cells [18,19]. In previous research studying the effects of 1,25-(OH)_2_D_3_ in AML differentiation, 1,25-(OH)_2_D_3_ was found to inhibit the entry of β-catenin protein into the nucleus by upregulating β-catenin–interacting protein 1 (ICAT) and then suppressing the Wnt/β-catenin signaling, inducing the differentiation of acute promyelocytic leukemia cells into a mononuclear line, with ICAT playing a pivotal role in this process [20]. Earlier investigations revealed that ICAT inhibits glioblastoma proliferation and suppresses colorectal cancer progression by targeting the Wnt/β-catenin signaling [21,22]. Nevertheless, the precise mechanism by which 1,25-(OH)_2_D_3_ and ICAT affect AML cell stemness remains largely unknown.

In this study, HL-60 and NB4 cells underwent treatment with 1,25(OH)_2_D_3_ to assess its impact on cell proliferation, differentiation, and the expression of markers associated with stemness. The investigation aimed to uncover how 1,25(OH)_2_D_3_ influences the stem cell characteristics of AML cells. Our data demonstrated that 1,25(OH)_2_D_3_ inhibited the stemness of HL-60 and NB4 cells, providing evidence supporting the use of 1,25-(OH)_2_D_3_ in leukemia treatment. Additionally, the experimental data from prior investigations revealed that ICAT protein expression was increased in HL-60 cells following exposure to 1,25(OH)_2_D_3_. Leveraging CRISPR/Cas9 gene-editing technology alongside lentivirus transfection, we successfully engineered HL-60 cell models featuring ICAT knockout or overexpression. Subsequently, we demonstrated that 1,25(OH)_2_D_3_ suppresses stemness through the Wnt/β-catenin signaling via a mechanism mediated by ICAT. This provides novel insights into the potential mechanisms of current antitumor therapy in leukemia.

## Materials and Methods

### Cell culture

NB4 cells were purchased from Wuxi Newgain Biotechnology Co., Ltd. (Wuxi, China), and HL-60 cells were procured from the Chinese Academy of Sciences. HL60 cells were cultured in IMDM medium (SH30228.01, Cytiva, CO, USA), with 20% fetal bovine serum (Gibco, Grand Island, USA) and 1% penicillin-streptomycin (Gibco, Grand Island, USA). NB4 cells were cultured in RPMI-1640 medium (CGM112.05, Cellmax, Guangzhou, China) with 10% fetal bovine serum (Gibco, Grand Island, USA) and 1% penicillin-streptomycin (Gibco, Grand Island, USA). All cells were maintained in a 37°C, 5% CO_2_ cell culture incubator. 1,25-(OH)_2_D_3_ (D1530, Sigma-Aldrich, St. Louis, MO, USA) was dissolved in absolute ethyl alcohol and stored at −20°C at a concentration of 1 × 10^−3^ M/L. The concentration of 1,25-(OH)_2_D_3_ in the drug medium was 1 × 10^−7^ M/L.

### CCK-8 assay

Cells were seeded at a density of 10^4^ cells in 96-well plates. Cells in the drug group were incubated with 1 × 10^−7^ M 1,25-(OH)_2_D_3_, and cells in the control group were untreated. All other conditions were identical between the groups. Twenty microliters of CCK-8 reagent (Beyotime, Shanghai, China) were added to each well at the specified time point (0, 1 day, 2 day, and 3 day), followed by incubation for 2.5 h, and the absorbance at 450 nm was measured in a spectrophotometer (Bio-Rad, Hercules, CA, USA) to calculate the relative growth rate of the cells.

### Immunohistochemical assay

Cells from both the control and experimental groups were collected at 72 h for Wright–Giemsa staining (BioVision, Guangzhou, China). Esterase staining was performed using the Acid α-Naphthyl Acetate Esterase Staining Kit (G2390, Solarbio, Guangzhou, China) [23], and NaF inhibition assays were conducted to observe cellular morphology using a BX-63 upright microscope (Olympus, Tokyo, Japan).

### CD14 detection by flow cytometry

Approximately 2 × 10^5^/mL cells were exposed to 1,25-(OH)_2_D_3_ (10^−7^ M, as a positive control) for 72 h. Cells were washed for twice by pre-cold 1× PBS. Then, incubated with anti-CD14 antibody (Becton Dickinson, NJ, USA) in the dark. After 30 min, cells were washed by pre-cold PBS and detected with flow cytometry (CytoFLEX S, Beckman-Coulter, CA, USA) [24].

### Cell sphere formation assay

First, 0.5 × 10^3^ cells were seeded into ultra-low attachment 24-well plates. Starting from day 0, 200 µL of sphere-specific culture medium was added, and 200 µl of sphere-specific culture medium was added again on days 3, 6, 9, and 12. Photographic documentation and quantification of tumor spheres (diameter > 30 μm) were conducted on days 9, 12, and 15 [25].

### Western blot blotting

Total proteins was extracted using RIPA lysis buffer (C1053, Applygen, Beijing, China) containing protease inhibitors (4693132001, Roche, Basel, Switzerland), and the protein concentration was determined by the bicinchoninic acid assay (BIV-K812-1000, BioVision). Proteins (30 µg/well) were separated by 12% SDS-PAGE and then transferred to a PVDF membrane (ISEQ00010, Millipore, Danvers, MA, USA). Membranes were sealed with 5% skim milk for 0.5 h and incubated with the following primary antibodies at 4°C overnight: anti-β-tubulin (AF1216, 1:5000, Beyotime), anti-SOX2 (ab97959, 1:2000, Abcam, Cambridge, UK), anti-OCT4 (ab179800, 1:2000, Abcam), anti-Nanog (4903S, 1:2000, CST, Danvers, MA, USA), and anti-c-Myc (GTX10825, 1:1000, GeneTex, SoCal, UK). Subsequently, the membranes were incubated for 1.5 h at 24°C with Alexa Fluor 488B-labeled anti-rabbit IgG (ab6721, 1:5000, Abcam). Immobilon Western Chemiluminescent HRP Substrate (WBKLS0500, Millipore, MA, USA) was used to detect the blots using a chemiluminescence imager (Fusion SoloS. EDGE, VILBER, Paris, France). The intensity was analyzed using Image Lab™ software (Bio-Rad).

### Quantitative real-time polymerase chain reaction (qRT-PCR)

The experimental and control groups of cells were subjected to total RNA extraction with TRIzol reagent (15596026, Life Technologies, South San Francisco, CA, USA). Subsequently, cDNA synthesis was performed following the manufacturer’s instructions using the HiScript® III RT SuperMix for qPCR (+gDNA wiper) kit (R223-01, Vazyme, Nanjing, China). The sequences of primers are shown in Table 1. qRT-PCR was conducted using PowerUp™ SYBR™ Green Master Mix (A25742, Applied Biosystems, Waltham, MA, USA) and the ABI PCR System (Applied Biosystems). The thermocycling parameters were as follows: 50°C for 120 s; 95°C for 120 s; and 40 cycles of 95°C for 15 s and 60°C for 60 s. The dissolution curve protocol was 95°C for 15 s, 60°C for 60 s, and 95°C for 15 s. Relative gene expression levels were calculated by the 2^−ΔΔCq^ method [26]. GAPDH was applied as a normalization control.

**Table 1:** Representative gene primers used to perform qRT-PCR

Gene	Primer sequences (5′→3′)
OCT4	F: 5′-GACAGGGGGAGGGGAGGAGCTAGG-3′
	R: 5′-CTTCCCTCCAACCAGTTGCCCCAAAC-3′
nanog	F: 5′-CAGCCCAGATTCTTCCACCAGTCCC-3′
	R: 5′-CGGAAGCGTTCCCAGTCGGGTTCACC-3′
SOX2	F:5′-GGGAAATGGGAGGGGTGCAAAAGAGG-3′
	R: 5′-TTGCGTGAGTGTGGATGGGATTGGTG-3′
c-Myc	F: 5′-TGGAAAACCAGCCTCCC-3′
	R: 5′-CGTAGTCGAGGTCATAGTTC-3′
GAPDH	F: 5′-AAATCCCATCACCATCTTCCAG-3′
	R: 5′-AGGGGCCATCCACAGTCTTCT-3′

### CRISPR/Cas9 gene-editing and lentivirus infection techniques

The human CTNNBIP1 (ICAT) gene was knocked out in HL-60 cells using CRISPR/Cas9 [27]. Homozygous ICAT gene knockout (Sh-HL-60) was confirmed by PCR and by sequencing electroporated single clones. The lentiviral vector LVEFS>KozakHumanCTNNBIP1CDS[NM_020248.3]CMV>EGFP/T2A/Puro was constructed, validated by PCR, and sequenced. 293T cells were infected with ICAT lentivirus, a control vector, and a helper plasmid for lentivirus packaging. The ICAT overexpression virus and control virus were then used to infect HL-60 cells, resulting in stably transfected ICAT-overexpressing cells (Gh-HL-60) and empty vector cells (EV-HL-60), as determined by screening with puromycin. Harvested cells were used for subsequent experiments.

### In vivo tumorigenesis assay

The animal experiments in the current study were approved and supervised by the Institutional Animal Care and Use Committee of Zhongshan People’s Hospital (No. K-2023-214). 54 (eighteen in each group) nude mice (6-week-old male NOD/SCID), which were purchased from GemPharmatech Co., Ltd., Nanjing, China, were maintained in pathogen-free facilities. All the animals were weighed and randomly into groups according to their weight using StudyDirector™ (3.1.399.19, Studylog System, Inc., S. San Francisco, CA, USA) Select the Matched distribution method for grouping. Approximately 106 Sh-HL60, Gh-HL60, and HL60 cells were injected subcutaneously into the right flank of mice (eighteen mice in each group) for tumorigenesis. When the average tumor volume reached 100 mm^3^, mice were randomly assigned to two groups with 3 mice per group: the treatment (1,25-(OH)_2_D_3_) and control (PBS) groups. Mice in the treatment group received 0.5 μg/kg 1,25-(OH)_2_D_3_ intraperitoneally twice a day for 2 weeks. Tumor size was measured twice a week. Tumour volumes were calculated according to the formula (width2 × length)/2. The mice were humanely killed, and the weight of the excised tumor was measured.

### Statistical analysis

All data in this study were generated from three independent experiments. Statistical analyses were conducted with the GraphPad Prism 7.0 software. Differences between the two groups were assessed by the two-tailed Student’s *t*-test. Differences between before and after treatment were assessed by one-way analysis of variance (ANOVA). *p* < 0.05 indicated statistical significance.

## Results

### Antiproliferative and monocytic differentiation-inducing effects of 1,25-(OH)_2_D_3_ I AML cells

**Figure 1:**
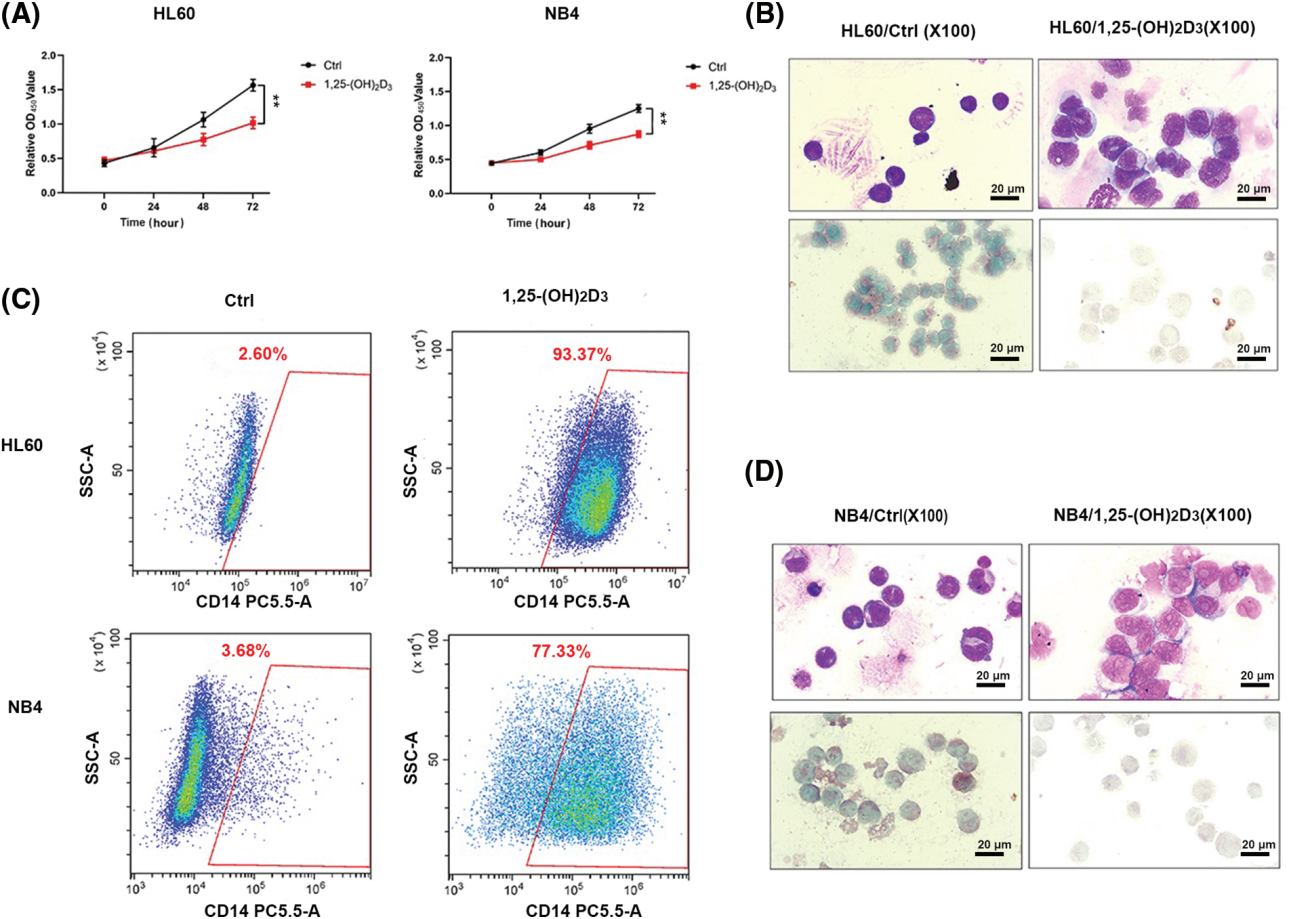
Antiproliferative and monocytic differentiation-inducing effects of 1,25-(OH)_2_D_3_ on AML cells. (A) The proliferation of HL-60 and NB4 cells following 1,25-(OH)_2_D_3_ treatment for 0, 24, 48, and 72 h. The results are presented as the mean ± SD of triplicate experiments. **p < 0.01. (B) Representative images of Wright–Giemsa staining, esterase staining, and NaF inhibition experiments in untreated and drug-treated HL-60 cells. (C) Representative flow cytometry data depicting the percentage of cells that were positive for the surface antigen CD14 in untreated and drug-treated HL-60 and NB4 cells. (D) Representative images of Wright–Giemsa staining, esterase staining, and NaF inhibition experiments in untreated and drug-treated NB4 cells.

We used the CCK-8 assay to validate the suppressive effects of 1,25-(OH)_2_D_3_ on the proliferation of HL-60 and NB4 cells (Fig. 1A). We also employed an immunohistochemical assay to elucidate the monocytic lineage differentiation induced by 1,25-(OH)_2_D_3_ in NB4 and HL-60 cells (Fig. 1B,D). The expression of the monocytic differentiation marker CD14 was remarkably upregulated after treatment (Fig. 1C). The results indicated that 1,25-(OH)_2_D_3_ significantly inhibits cell proliferation in HL-60 and NB4 cells while inducing their differentiation toward the monocytic lineage.

### Inhibition of stemness by 1,25-(OH)_2_D_3_ in AML cells

**Figure 2:**
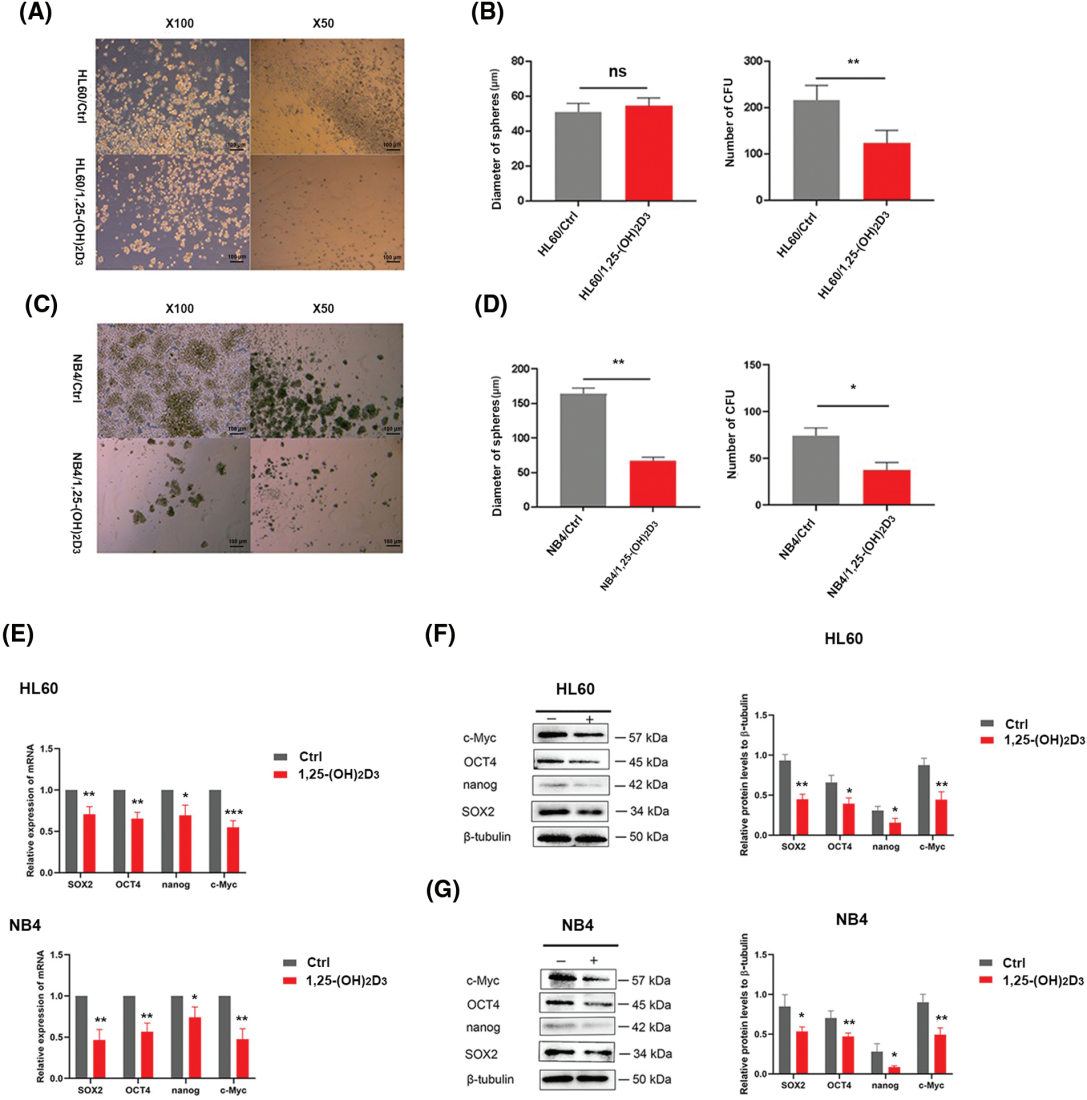
Inhibition of stemness by 1,25-(OH)_2_D_3_ in AML cells. (A) Stemness of HL-60 cells treated with or without 1,25-(OH)_2_D_3_ (Ctrl) was analyzed by the sphere formation assay (×50 and ×100 magnification). (B) Quantification and statistical analysis of the data in A. (C) Stemness of NB4 cells treated with or without 1,25-(OH)_2_D_3_ (Ctrl) was analyzed by the sphere formation assay (×50 and ×100 magnification). (D) Quantification and statistical analysis of the data in C. (E) qRT-PCR of SOX2, OCT4, Nanog, and c-Myc expression. (F) Protein expression of SOX2, OCT4, Nanog, and c-Myc after treatment with or without 1,25-(OH)_2_D_3_ for 72 h in HL-60 cells was analyzed by western blotting. (G) Protein expression of OCT4, SOX2, c-Myc, and Nanog after treatment with or without 1,25-(OH)_2_D_3_ for 72 h in NB4 cells was verified by western blot. ^ns^*p* > 0.05, *p < 0.05, **p < 0.01, ***p < 0.001.

We also conducted a cell sphere formation assay to further determine the effects of 1,25-(OH)_2_D_3_ on the stemness of HL-60 and NB4 cells. 1,25-(OH)_2_D_3_ suppressed sphere formation by these cells, as evidenced by reductions in the number (Fig. 2A,B) and diameter of spheres (Fig. 2C,D). Regarding stemness marker gene expression, the expression of OCT4, Nanog, SOX2, and c-Myc was inhibited by 1,25-(OH)_2_D_3_ at the protein (Fig. 2E) and mRNA levels (Fig. 2F,G). These results indicated that 1,25-(OH)_2_D_3_ significantly decreased the stemness of HL-60 and NB4 leukemia cells.

### Validation of stable HL-60 strains with ICAT gene knockout or overexpression

**Figure 3:**
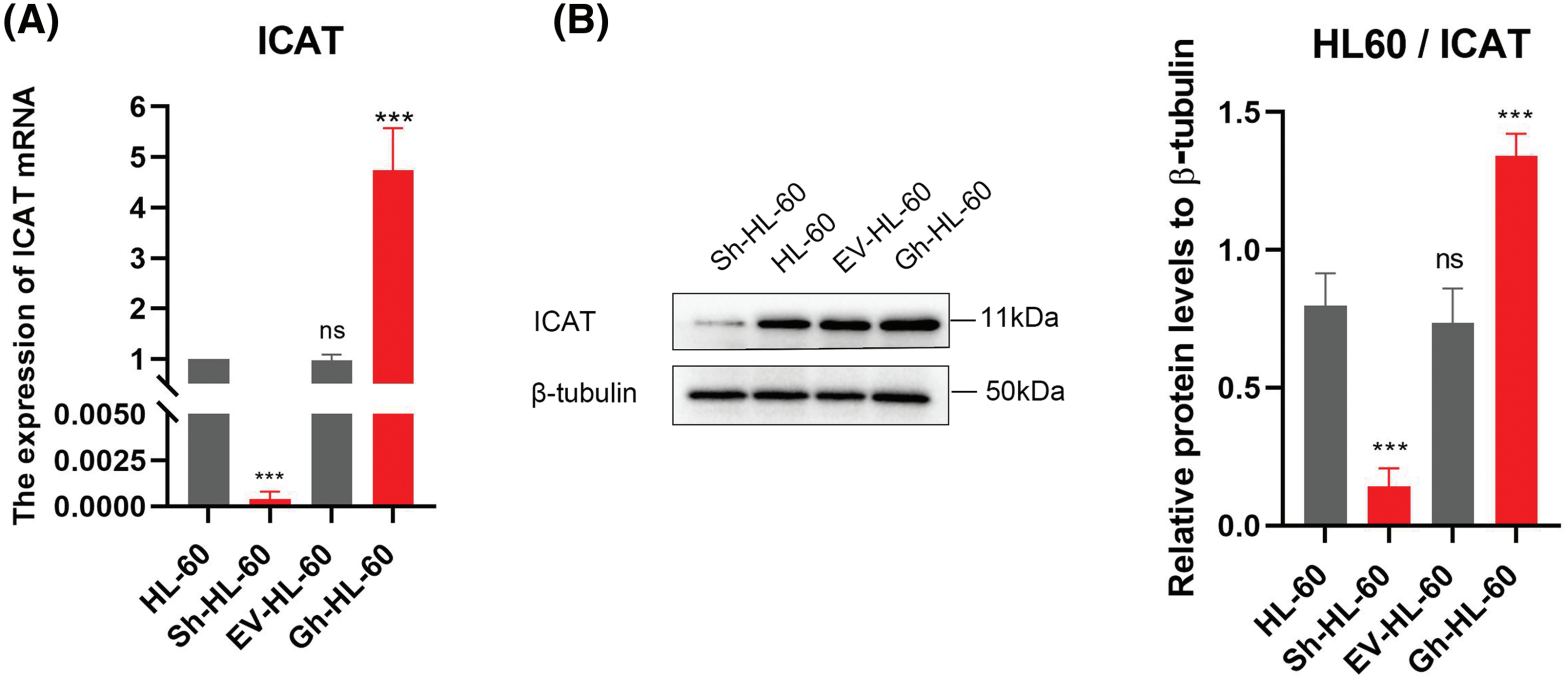
Stable HL-60 cells were identified with both ICAT gene knockout and overexpression. (A) Quantitative real-time PCR was conducted to assess ICAT expression in these stable HL-60 transfectants. (B) Western blotting analysis was performed to examine ICAT expression in the stable HL-60 cells. Results represent the mean ± SD of triplicate experiments. ^ns^*p* > 0.05, ***p < 0.001.

**Figure A1:**
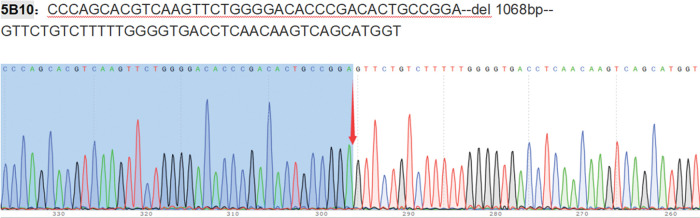
IACT knockout monoclonal sequencing results. IACT knockout HL-60 cells monoclonal sequencing results.

HL-60 cells stably transfected with ICAT gene knockout and ICAT gene overexpression constructs were successfully generated and verified by qRT-PCR and western blotting, as presented in Fig. 3A,B. The sequencing results of ICAT gene knockout and overexpression vector construction are provided in the Appendix A (Fig. A1 and Table A1).

### Role of ICAT in the 1,25-(OH)_2_D_3_-mediated inhibition of AML cells proliferation and induction of monocytic differentiation

**Figure 4:**
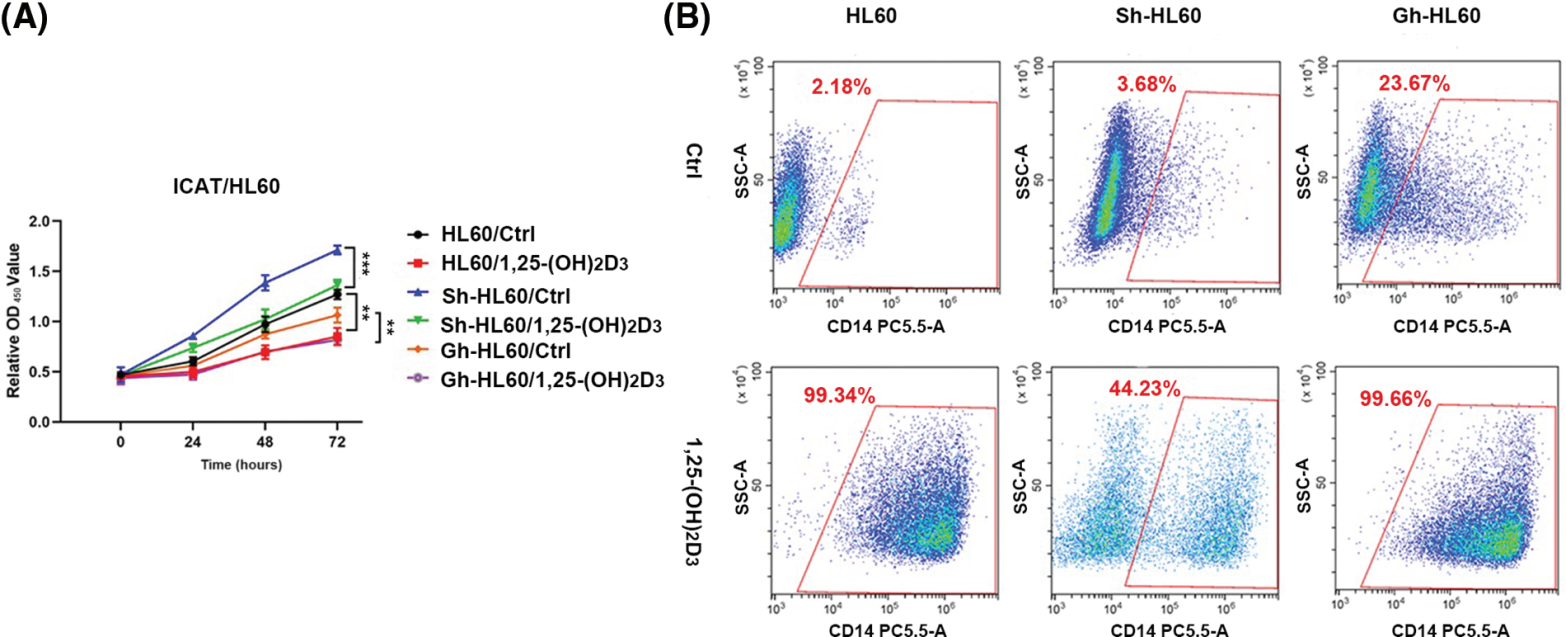
ICAT expression’s involvement in the inhibition of AML cell proliferation and the promotion of monocytic differentiation by 1,25-(OH)_2_D_3_ was investigated. (A) HL-60, Sh-HL-60, and Gh-HL-60 cell proliferation was monitored at 0, 24, 48, and 72 h post 1,25-(OH)_2_D_3_ treatment. Data represent the mean ± SD of triplicate experiments. **p < 0.01, ***p < 0.001. (B) Flow cytometry analysis demonstrated the percentage of CD14-positive cells among treated and untreated HL-60, Sh-HL-60, and Gh-HL-60 cells.

The results of the CCK-8 assay indicated 1,25-(OH)_2_D_3_ inhibited the proliferation of HL-60 cells, and this effect was lessened by ICAT knockout (Fig. 4A). Flow cytometry revealed 1,25-(OH)_2_D_3_ induced mononuclear differentiation, and this effect was weakened by ICAT knockout and increased by more than 20% by ICAT overexpression (Fig. 4B). These findings indicate that 1,25-(OH)_2_D_3_ may influence HL-60 cell differentiation through ICAT expression.

### Enhancement of the 1,25-(OH)_2_D_3_-induced inhibition of AML cells sphere formation by ICAT expression

**Figure 5:**
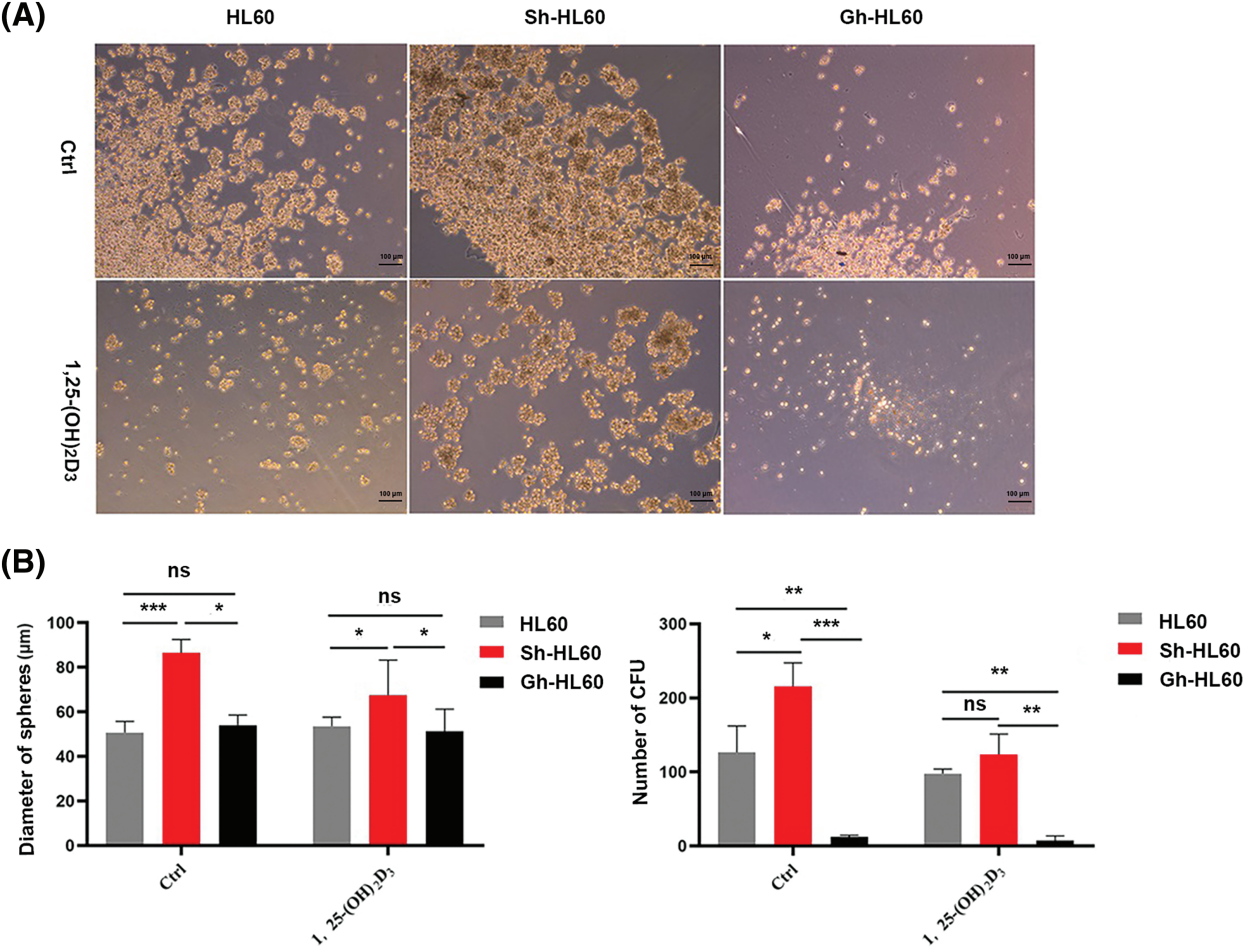
ICAT expression enhanced the inhibitory effect of 1,25-(OH)_2_D_3_ on AML cell sphere formation. (A) Stemness of HL-60, Sh-HL-60, and Gh-HL-60 cells treated with or without 1,25-(OH)_2_D_3_ (Ctrl) was analyzed by the sphere formation assay (×100 magnification). (B) Quantification and statistical analysis of the data in A. Results represent the mean ± SD of triplicate experiments. ^ns^*p* > 0.05, *p < 0.05, **p < 0.01, ***p < 0.001.

We performed cell sphere formation experiments to examine the role of ICAT expression in the inhibition of HL-60 cell sphere formation by 1,25-(OH)_2_D_3_. Our results indicated that, in untreated control cells, knockdown of ICAT alone enhanced sphere formation by HL-60 cells, whereas overexpression of ICAT alone inhibited their spheroidization. Meanwhile, in cells incubated with 1,25-(OH)_2_D_3_, ICAT overexpression enhanced its inhibitory effect, whereas 1,25-(OH)_2_D_3_ could not effectively inhibit the spheroidization of HL-60 cells after ICAT knockout, as evidenced by the changes in the number and diameter of spheroids (Fig. 5A,B).

### Downregulation of stemness markers by 1,25-(OH)_2_D_3_ through ICAT expression in AML cells

**Figure 6:**
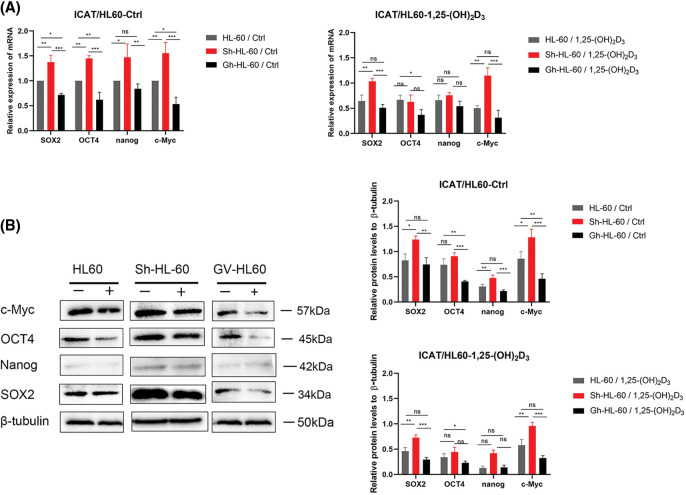
Downregulation of stem cell markers by 1,25-(OH)_2_D_3_ through ICAT expression in AML cells. (A) qRT-PCR of SOX2, OCT4, Nanog, and c-Myc expression in HL-60, Sh-HL-60, and Gh-HL-60 cells after treatment with or without 1,25-(OH)_2_D_3_ for 72 h. (B) Protein expression of SOX2, OCT4, Nanog, and c-Myc after treatment with or without 1,25-(OH)_2_D_3_ for 72 h in HL-60, Sh-HL-60, and Gh-HL-60 cells was analyzed by western blotting. Results represent the mean ± SD of triplicate experiments. ^ns^*p* > 0.05, *p < 0.05, **p < 0.01, ***p < 0.001.

We assessed stemness-related gene expression (SOX2, Nanog, OCT4, c-Myc) in AML cells using qRT-PCR. ICAT knockout lessened the inhibitory effects of 1,25-(OH)_2_D_3_ on SOX2 and c-Myc expression, whereas these effects were enhanced by ICAT overexpression. In addition, ICAT overexpression enhanced the effects of 1,25-(OH)_2_D_3_ on OCT4 expression (Fig. 6A). The results of western blotting aligned with those of qRT-PCR (Fig. 6B), indicating that ICAT promoted the downregulation of stemness markers in AML cells treated with 1,25-(OH)_2_D_3_. This suggests that 1,25-(OH)_2_D_3_ inhibits AML cell stemness by modulating ICAT, and ICAT alone can suppress stemness characteristics.

### Inhibition of xenograft growth by 1,25-(OH)_2_D_3_ and ICAT expression in vivo

**Figure 7:**
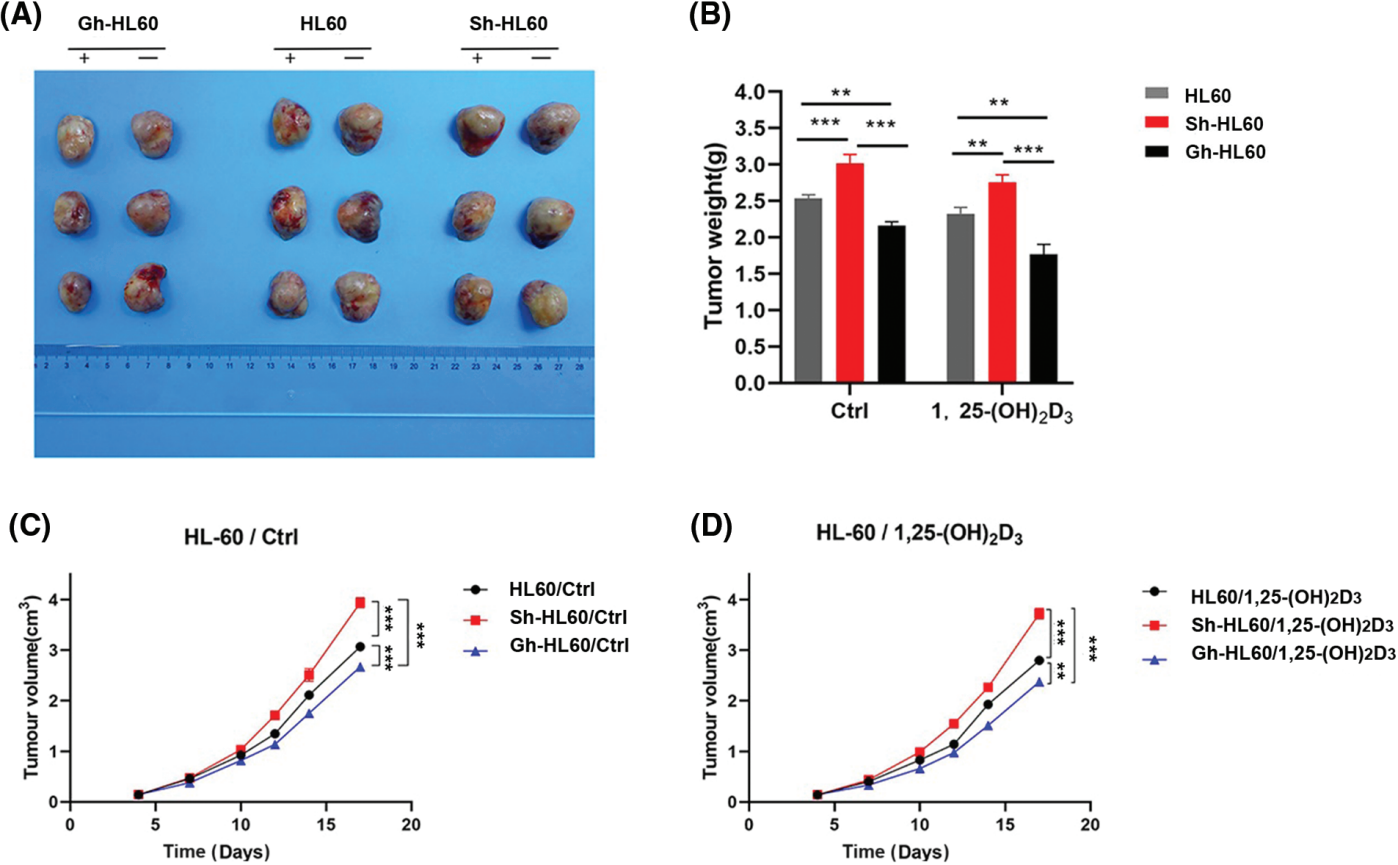
Inhibition of xenograft tumor by 1,25-(OH)_2_D_3_ and ICAT expression *in vivo*. (A) Photographs of tumors xenografts. (B) The effects of 1,25-(OH)_2_D_3_ and ICAT expression on tumor weight. (C) The corresponding tumor volumes in the untreated group at the indicated times are presented. (D) The corresponding tumor volumes after 1,25-(OH)_2_D_3_ injection on the indicated days. **p < 0.01, ***p < 0.001.

We conducted an *in vivo* tumor growth study using HL-60, Sh-HL-60, and Gh-HL-60 xenograft mouse models. 1,25-(OH)_2_D_3_ treatment significantly reduced tumor size and weight in mice. In the untreated control group, overexpression of ICAT reduced the volume and weight of xenograft tumors in the HL-60 model, whereas knockdown of ICAT increased tumor volume and weight (Fig. 7A–C). In the 1,25-(OH)_2_D_3_ treatment group, overexpression of ICAT enhanced the tumor-suppressing effect of 1,25-(OH)_2_D_3_, whereas this effect was attenuated by ICAT knockout (Fig. 7B,D).

## Discussion

Cell stemness is defined by its quiescent state, pluripotent nature, and ability for long-term self-renewal [22]. It plays critical roles in leukemia initiation, progression, and relapse [28], but the key determinants of leukemia cell stemness are poorly understood [29]. Therefore, targeting leukemia cell stemness could be a promising strategy for improving the efficacy of leukemia treatment [30]. 1,25-(OH)_2_D_3_ is the most biologically active vitamin D metabolite [31]. Upon binding with its receptor, it initiates the translocation of the receptor complex to the nucleus, where it interacts with the genome, regulating the expression of over 1200 genes [32]. Studies have illustrated that immune cells, such as monocytes, dendritic cells, lymphocytes, and macrophages, express the vitamin D receptor. 1,25-(OH)_2_D_3_ can activate immune cells, such as T and B cells, macrophages, and dendritic cells, to regulate immunity and increase the production of antimicrobial peptides and neutralizing antibodies [33–35]. In addition, studies have revealed that 1,25-(OH)_2_D_3_ acts as an efficient anticancer agent through several signaling pathways [36–40]. Recent data suggest that 1,25-(OH)_2_D_3_ plays a regulatory function in both normal and cancerous stem cells, exerting an inhibitory effect on stem-like properties across diverse tumor types including hepatocellular carcinoma, breast, colorectal, prostate, and gastric cancer cells [36,41–45]. However, the effects of 1,25-(OH)_2_D_3_ on the stemness of AML cells has not been well studied. In this study, we investigated the effects of 1,25-(OH)_2_D_3_ on the stemness of AML cells. The experimental results provided evidence that 1,25-(OH)_2_D_3_ may directly inhibit the stemness of AML cells, and the compound suppressed the proliferation of NB4 and HL-60 cells and induced their differentiation into monocytes. This is consistent with previous studies suggesting that 1,25-(OH)_2_D_3_ can stimulate myeloid stem cells to preferentially differentiate into monocytes and macrophages [46]. In addition, the present results revealed that 1,25-(OH)_2_D_3_ can also suppress sphere formation by HL-60 and NB4 cells and suppress the expression of the stemness markers SOX2, Nanog, OCT4, and c-Myc in HL-60 and NB4 cells. The results indicated that 1,25-(OH)_2_D_3_ can inhibit the stemness of AML cells, as previously observed in breast cancer, colorectal cancer, and other tumors.

In our previous studies examining the role of ICAT in AML differentiation, we found that the entry of β-catenin protein into the nucleus was inhibited by upregulating ICAT expression, and then inhibition of the Wnt/β-catenin signaling pathway induced the differentiation of HL-60 acute promyelocytic leukemia cells into a mononuclear line [20]. ICAT is a 9-kDa polypeptide that plays a negative role in the Wnt/β-catenin signaling by inhibiting β-catenin nuclear signaling via binding to β-catenin and competing with the transcription factor T cell factor [47,48]. ICAT is a key protein in the Wnt/β-catenin signaling that negatively modulates β-catenin co-transcriptional activity [48,49]. As an important molecule of the Wnt signaling pathway, ICAT sustains stemness by maintaining multipotency in certain cell types [50]. Activation of the Wnt/β-catenin signaling is one of the important mechanisms of tumorigenesis [51]. To further elucidate the function of ICAT in the 1,25-(OH)_2_D_3_-mediated inhibition of AML cell stemness, we knocked out and overexpressed the ICAT gene in HL-60 cells and analyzed its role by western blotting, qRT-PCR, and cell sphere formation assays. In untreated cells, knockout of ICAT alone enhanced the stemness of HL-60 cells, while upregulation of ICAT inhibited the stemness of these cells. In the 1,25-(OH)_2_D_3_ treatment group, ICAT overexpression enhanced the stemness-inhibiting effect of 1,25-(OH)_2_D_3_ in AML cells, whereas ICAT knockout weakened this inhibitory effect. These results suggested that 1,25-(OH)_2_D_3_ suppresses stemness through the Wnt/β-catenin signaling through a mechanism mediated by ICAT protein. Moreover, we validated these results *in vivo* in NOD/SCID mice. Previous studies illustrated that 1,25-(OH)_2_D_3_ suppresses cancer cells proliferation and promotes their differentiation, primarily by antagonizing TGF-β, Wnt/β-catenin, and EGF signaling pathways [6]. However, 1,25-(OH)_2_D_3_ interacts with the vitamin D receptor which binds to specific DNA sequences known as vitamin D response elements, thereby controlling the expression of interconnected downstream genes [52].

Recent research has shown that decreased ICAT expression correlates with unfavorable disease-free and overall survival outcomes in AML, indicating that ICAT is closely involved in AML progression [50]. In the current study, our initial focus was on understanding the role of ICAT in the suppression of AML cell stemness by 1,25-(OH)_2_D_3_. Our findings revealed that 1,25-(OH)_2_D_3_ reduces AML cell stemness by targeting the Wnt/β-catenin signaling, a process facilitated by ICAT protein. Nevertheless, there were certain limitations in our research. Notably, while the dosage of 1,25-(OH)_2_D_3_ administered to nude mice was based on existing literature, the delay in tumor extraction resulted in larger tumor sizes.

## Conclusions

In summary, our study revealed that 1,25-(OH)_2_D_3_ suppresses the stem-like properties of AML cells, likely by modulating the Wnt/β-catenin signaling via ICAT. These findings offer fresh insights into how 1,25-(OH)_2_D_3_ may exert its anti-leukemic effects. Additionally, our observations suggest that ICAT plays a role in curtailing the stemness of AML cells, with its expression tightly linked to this characteristic. Therefore, further studies on the role and regulation of ICAT might reveal a novel therapeutic target for overcoming treatment resistance, improving prognosis, and preventing relapse in patients with AML.

## Data Availability

The original data in this study can be obtained from the corresponding author upon request.

## Appendix A

**Table A1:** Sequence of the final targeting vector of HL-60 cells overexpressing ICAT

	EFS promoter		CDS		EGFP/T2A/Puro			Sequence confirmed region			
1	AATGTA	GTCTTA		TGCAAT	ACTCTT	GTAGTC	TTGCAA	CATGGT	AACGAT	GAGTTA	GCAACA
61	TGCCTT	ACAAGG		AGAGAA	AAAGCA	CCGTGC	ATGCCG	ATTGGT	GGAAGT	AAGGTG	GTACGA
121	TCGTGC	CTTATT		AGGAAG	GCAACA	GACGGG	TCTGAC	ATGGAT	TGGACG	AACCAC	TGAATT
181	GCCGCA	TTGCAG		AGATAT	TGTATT	TAAGTG	CCTAGC	TCGATA	CATAAA	CGGGTC	TCTCTG
241	GTTAGA	CCAGAT		CTGAGC	CTGGGA	GCTCTC	TGGCTA	ACTAGG	GAACCC	ACTGCT	TAAGCC
301	TCAATA	AAGCTT		GCCTTG	AGTGCT	TCAAGT	AGTGTG	TGCCCG	TCTGTT	GTGTGA	CTCTGG
361	TAACTA	GAGATC		CCTCAG	ACCCTT	TTAGTC	AGTGTG	GAAAAT	CTCTAG	CAGTGG	CGCCCG
421	AACAGG	GACTTG		AAAGCG	AAAGGG	AAACCA	GAGGAG	CTCTCT	CGACGC	AGGACT	CGGCTT
481	GCTGAA	GCGCGC		ACGGCA	AGAGGC	GAGGGG	CGGCGA	CTGGTG	AGTACG	CCAAAA	ATTTTG
541	ACTAGC	GGAGGC		TAGAAG	GAGAGA	GATGGG	TGCGAG	AGCGTC	AGTATT	AAGCGG	GGGAGA
601	ATTAGA	TCGCGA		TGGGAA	AAAATT	CGGTTA	AGGCCA	GGGGGA	AAGAAA	AAATAT	AAATTA
661	AAACAT	ATAGTA		TGGGCA	AGCAGG	GAGCTA	GAACGA	TTCGCA	GTTAAT	CCTGGC	CTGTTA
721	GAAACA	TCAGAA		GGCTGT	AGACAA	ATACTG	GGACAG	CTACAA	CCATCC	CTTCAG	ACAGGA
781	TCAGAA	GAACTT		AGATCA	TTATAT	AATACA	GTAGCA	ACCCTC	TATTGT	GTGCAT	CAAAGG
841	ATAGAG	ATAAAA		GACACC	AAGGAA	GCTTTA	GACAAG	ATAGAG	GAAGAG	CAAAAC	AAAAGT
901	AAGACC	ACCGCA		CAGCAA	GCGGCC	GCTGAT	CTTCAG	ACCTGG	AGGAGG	AGATAT	GAGGGA
961	CAATTG	GAGAAG		TGAATT	ATATAA	ATATAA	AGTAGT	AAAAAT	TGAACC	ATTAGG	AGTAGC
1021	ACCCAC	CAAGGC		AAAGAG	AAGAGT	GGTGCA	GAGAGA	AAAAAG	AGCAGT	GGGAAT	AGGAGC
1081	TTTGTT	CCTTGG		GTTCTT	GGGAGC	AGCAGG	AAGCAC	TATGGG	CGCAGC	GTCAAT	GACGCT
1141	GACGGT	ACAGGC		CAGACA	ATTATT	GTCTGG	TATAGT	GCAGCA	GCAGAA	CAATTT	GCTGAG
1201	GGCTAT	TGAGGC		GCAACA	GCATCT	GTTGCA	ACTCAC	AGTCTG	GGGCAT	CAAGCA	GCTCCA
1261	GGCAAG	AATCCT		GGCTGT	GGAAAG	ATACCT	AAAGGA	TCAACA	GCTCCT	GGGGAT	TTGGGG
1321	TTGCTC	TGGAAA		ACTCAT	TTGCAC	CACTGC	TGTGCC	TTGGAA	TGCTAG	TTGGAG	TAATAA
1381	ATCTCT	GGAACA		GATTTG	GAATCA	CACGAC	CTGGAT	GGAGTG	GGACAG	AGAAAT	TAACAA
1441	TTACAC	AAGCTT		AATACA	CTCCTT	AATTGA	AGAATC	GCAAAA	CCAGCA	AGAAAA	GAATGA
1501	ACAAGA	ATTATT		GGAATT	AGATAA	ATGGGC	AAGTTT	GTGGAA	TTGGTT	TAACAT	AACAAA
1561	TTGGCT	GTGGTA		TATAAA	ATTATT	CATAAT	GATAGT	AGGAGG	CTTGGT	AGGTTT	AAGAAT
1621	AGTTTT	TGCTGT		ACTTTC	TATAGT	GAATAG	AGTTAG	GCAGGG	ATATTC	ACCATT	ATCGTT
1681	TCAGAC	CCACCT		CCCAAC	CCCGAG	GGGACC	CGACAG	GCCCGA	AGGAAT	AGAAGA	AGAAGG
1741	TGGAGA	GAGAGA		CAGAGA	CAGATC	CATTCG	ATTAGT	GAACGG	ATCTCG	ACGGTA	TCGCTA
1801	GCTTTT	AAAAGA		AAAGGG	GGGATT	GGGGGG	TACAGT	GCAGGG	GAAAGA	ATAGTA	GACATA
1861	ATAGCA	ACAGAC		ATACAA	ACTAAA	GAATTA	CAAAAA	CAAATT	ACAAAA	ATTCAA	AATTTT
1921	ACTAGT	GATTAT		CGGATT	GATAAT	CGAATT	CCACGT	GGGCTC	CGGTGC	CCGTCA	GTGGGC
1981	AGAGCG	CACATC		GCCCAC	AGTCCC	CGAGAA	GTTGGG	GGGAGG	GGTCGG	CAATTG	ATCCGG
2041	TGCCTA	GAGAAG		GTGGCG	CGGGGT	AAACTG	GGAAAG	TGATGT	CGTGTA	CTGGCT	CCGCCT
2101	TTTTCC	CGAGGG		TGGGGG	AGAACC	GTATAT	AAGTGC	AGTAGT	CGCCGT	GAACGT	TCTTTT
2161	TCGCAA	CGGGTT		TGCCGC	CAGAAC	ACAGGG	GATCCA	GCCACC	ATGAAC	CGCGAG	GGAGCT
2221	CCCGGG	AAGAGT		CCGGAG	GAGATG	TACATT	CAGCAG	AAGGTC	CGAGTG	CTGCTC	ATGCTG
2281	CGGAAG	ATGGGA		TCAAAC	CTGACA	GCCAGC	GAGGAG	GAGTTC	CTGCGC	ACCTAT	GCAGGG
2341	GTGGTC	AACAGC		CAGCTC	AGCCAG	CTGCCT	CCGCAC	TCCATC	GACCAG	GGTGCA	GAGGAC
2401	GTGGTG	ATGGCG		TTTTCC	AGGTCG	GAGACG	GAAGAC	CGGAGG	CAGTAG	GGCGCG	CCGATA
2461	ATCAAC	CTCTGG		ATTACA	AAATTT	GTGAAA	GATTGA	CTGGTA	TTCTTA	ACTATG	TTGCTC
2521	CTTTTA	CGCTAT		GTGGAT	ACGCTG	CTTTAA	TGCCTT	TGTATC	ATGCTA	TTGCTT	CCCGTA
2581	TGGCTT	TCATTT		TCTCCT	CCTTGT	ATAAAT	CCTGGT	TGCTGT	CTCTTT	ATGAGG	AGTTGT
2641	GGCCCG	TTGTCA		GGCAAC	GTGGCG	TGGTGT	GCACTG	TGTTTG	CTGACG	CAACCC	CCACTG
2701	GTTGGG	GCATTG		CCACCA	CCTGTC	AGCTCC	TTTCCG	GGACTT	TCGCTT	TCCCCC	TCCCTA
2761	TTGCCA	CGGCGG		AACTCA	TCGCCG	CCTGCC	TTGCCC	GCTGCT	GGACAG	GGGCTC	GGCTGT
2821	TGGGCA	CTGACA		ATTCCG	TGGTGT	TGTCGG	GGAAGC	TGACGT	CCTTTC	CATGGC	TGCTCG
2881	CCTGTG	TTGCCA		CCTGGA	TTCTGC	GCGGGA	CGTCCT	TCTGCT	ACGTCC	CTTCGG	CCCTCA
2941	ATCCAG	CGGACC		TTCCTT	CCCGCG	GCCTGC	TGCCGG	CTCTGC	GGCCTC	TTCCGC	GTCTTC
3001	GCCTTC	GCCCTC		AGACGA	GTCGGA	TCTCCC	TTTGGG	CCGCCT	CCCCGC	ATCGGG	AATTCC
3061	CGCGGT	TCGAAC		GCGATC	GCGCGT	TGACAT	TGATTA	TTGACT	AGTTAT	TAATAG	TAATCA
3121	ATTACG	GGGTCA		TTAGTT	CATAGC	CCATAT	ATGGAG	TTCCGC	GTTACA	TAACTT	ACGGTA
3181	AATGGC	CCGCCT		GGCTGA	CCGCCC	AACGAC	CCCCGC	CCATTG	ACGTCA	ATAATG	ACGTAT
3241	GTTCCC	ATAGTA		ACGCCA	ATAGGG	ACTTTC	CATTGA	CGTCAA	TGGGTG	GAGTAT	TTACGG
3301	TAAACT	GCCCAC		TTGGCA	GTACAT	CAAGTG	TATCAT	ATGCCA	AGTACG	CCCCCT	ATTGAC
3361	GTCAAT	GACGGT		AAATGG	CCCGCC	TGGCAT	TATGCC	CAGTAC	ATGACC	TTATGG	GACTTT
3421	CCTACT	TGGCAG		TACATC	TACGTA	TTAGTC	ATCGCT	ATTACC	ATGGTG	ATGCGG	TTTTGG
3481	CAGTAC	ATCAAT		GGGCGT	GGATAG	CGGTTT	GACTCA	CGGGGA	TTTCCA	AGTCTC	CACCCC
3541	ATTGAC	GTCAAT		GGGAGT	TTGTTT	TGGCAC	CAAAAT	CAACGG	GACTTT	CCAAAA	TGTCGT
3601	AACAAC	TCCGCC		CCATTG	ACGCAA	ATGGGC	GGTAGG	CGTGTA	CGGTGG	GAGGTC	TATATA
3661	AGCAGA	GCTCTC		TGGCTA	ACTAGA	GAACCC	ACTGCG	CCACCA	TGGTGA	GCAAGG	GCGAGG
3721	AGCTGT	TCACCG		GGGTGG	TGCCCA	TCCTGG	TCGAGC	TGGACG	GCGACG	TAAACG	GCCACA
3781	AGTTCA	GCGTGT		CCGGCG	AGGGCG	AGGGCG	ATGCCA	CCTACG	GCAAGC	TGACCC	TGAAGT
3841	TCATCT	GCACCA		CCGGCA	AGCTGC	CCGTGC	CCTGGC	CCACCC	TCGTGA	CCACCC	TGACCT
3901	ACGGCG	TGCAGT		GCTTCA	GCCGCT	ACCCCG	ACCACA	TGAAGC	AGCACG	ACTTCT	TCAAGT
3961	CCGCCA	TGCCCG		AAGGCT	ACGTCC	AGGAGC	GCACCA	TCTTCT	TCAAGG	ACGACG	GCAACT
4021	ACAAGA	CCCGCG		CCGAGG	TGAAGT	TCGAGG	GCGACA	CCCTGG	TGAACC	GCATCG	AGCTGA
4081	AGGGCA	TCGACT		TCAAGG	AGGACG	GCAACA	TCCTGG	GGCACA	AGCTGG	AGTACA	ACTACA
4141	ACAGCC	ACAACG		TCTATA	TCATGG	CCGACA	AGCAGA	AGAACG	GCATCA	AGGTGA	ACTTCA
4201	AGATCC	GCCACA		ACATCG	AGGACG	GCAGCG	TGCAGC	TCGCCG	ACCACT	ACCAGC	AGAACA
4261	CCCCCA	TCGGCG		ACGGCC	CCGTGC	TGCTGC	CCGACA	ACCACT	ACCTGA	GCACCC	AGTCCG
4321	CCCTGA	GCAAAG		ACCCCA	ACGAGA	AGCGCG	ATCACA	TGGTCC	TGCTGG	AGTTCG	TGACCG
4381	CCGCCG	GGATCA		CTCTCG	GCATGG	ACGAGC	TGTACA	AGGGCT	CCGGAG	AGGGCA	GGGGAA
4441	GTCTTC	TAACAT		GCGGGG	ACGTGG	AGGAAA	ATCCCG	GCCCCA	TGACCG	AGTACA	AGCCCA
4501	CGGTGC	GCCTCG		CCACCC	GCGACG	ACGTCC	CCAGGG	CCGTAC	GCACCC	TCGCCG	CCGCGT
4561	TCGCCG	ACTACC		CCGCCA	CGCGCC	ACACCG	TCGATC	CGGACC	GCCACA	TCGAGC	GGGTCA
4621	CCGAGC	TGCAAG		AACTCT	TCCTCA	CGCGCG	TCGGGC	TCGACA	TCGGCA	AGGTGT	GGGTCG
4681	CGGACG	ACGGCG		CCGCGG	TGGCGG	TCTGGA	CCACGC	CGGAGA	GCGTCG	AAGCGG	GGGCGG
4741	TGTTCG	CCGAGA		TCGGCC	CGCGCA	TGGCCG	AGTTGA	GCGGTT	CCCGGC	TGGCCG	CGCAGC
4801	AACAGA	TGGAAG		GCCTCC	TGGCGC	CGCACC	GGCCCA	AGGAGC	CCGCGT	GGTTCC	TGGCCA
4861	CCGTCG	GCGTCT		CGCCCG	ACCACC	AGGGCA	AGGGTC	TGGGCA	GCGCCG	TCGTGC	TCCCCG
4921	GAGTGG	AGGCGG		CCGAGC	GCGCCG	GGGTGC	CCGCCT	TCCTGG	AGACCT	CCGCGC	CCCGCA
4981	ACCTCC	CCTTCT		ACGAGC	GGCTCG	GCTTCA	CCGTCA	CCGCCG	ACGTCG	AGGTGC	CCGAAG
5041	GACCGC	GCACCT		GGTGCA	TGACCC	GCAAGC	CCGGTG	CCTGAG	GTACCT	TTAAGA	CCAATG
5101	ACTTAC	AAGGCA		GCTGTA	GATCTT	AGCCAC	TTTTTA	AAAGAA	AAGGGG	GGACTG	GAAGGG
5161	CTAATT	CACTCC		CAACGA	AGACAA	GATCTG	CTTTTT	GCTTGT	ACTGGG	TCTCTC	TGGTTA
5221	GACCAG	ATCTGA		GCCTGG	GAGCTC	TCTGGC	TAACTA	GGGAAC	CCACTG	CTTAAG	CCTCAA
5281	TAAAGC	TTGCCT		TGAGTG	CTTCAA	GTAGTG	TGTGCC	CGTCTG	TTGTGT	GACTCT	GGTAAC
5341	TAGAGA	TCCCTC		AGACCC	TTTTAG	TCAGTG	TGGAAA	ATCTCT	AGCAGT	AGTAGT	TCATGT
5401	CATCTT	ATTATT		CAGTAT	TTATAA	CTTGCA	AAGAAA	TGAATA	TCAGAG	AGTGAG	AGGAAC
5461	TTGTTT	ATTGCA		GCTTAT	AATGGT	TACAAA	TAAAGC	AATAGC	ATCACA	AATTTC	ACAAAT
5521	AAAGCA	TTTTTT		TCACTG	CATTCT	AGTTGT	GGTTTG	TCCAAA	CTCATC	AATGTA	TCTTAT
5581	CATGTC	TGGCTC		TAGCTA	TCCCGC	CCCTAA	CTCCGC	CCATCC	CGCCCC	TAACTC	CGCCCA
5641	GTTCCG	CCCATT		CTCCGC	CCCATG	GCTGAC	TAATTT	TTTTTA	TTTATG	CAGAGG	CCGAGG
5701	CCGCCT	CGGCCT		CTGAGC	TATTCC	AGAAGT	AGTGAG	GAGGCT	TTTTTG	GAGGCC	TAGGGA
5761	CGTACC	CAATTC		GCCCTA	TAGTGA	GTCGTA	TTACGC	GCGCTC	ACTGGC	CGTCGT	TTTACA
5821	ACGTCG	TGACTG		GGAAAA	CCCTGG	CGTTAC	CCAACT	TAATCG	CCTTGC	AGCACA	TCCCCC
5881	TTTCGC	CAGCTG		GCGTAA	TAGCGA	AGAGGC	CCGCAC	CGATCG	CCCTTC	CCAACA	GTTGCG
5941	CAGCCT	GAATGG		CGAATG	GGACGC	GCCCTG	TAGCGG	CGCATT	AAGCGC	GGCGGG	TGTGGT
6001	GGTTAC	GCGCAG		CGTGAC	CGCTAC	ACTTGC	CAGCGC	CCTAGC	GCCCGC	TCCTTT	CGCTTT
6061	CTTCCC	TTCCTT		TCTCGC	CACGTT	CGCCGG	CTTTCC	CCGTCA	AGCTCT	AAATCG	GGGGCT
6121	CCCTTT	AGGGTT		CCGATT	TAGTGC	TTTACG	GCACCT	CGACCC	CAAAAA	ACTTGA	TTAGGG
6181	TGATGG	TTCACG		TAGTGG	GCCATC	GCCCTG	ATAGAC	GGTTTT	TCGCCC	TTTGAC	GTTGGA
6241	GTCCAC	GTTCTT		TAATAG	TGGACT	CTTGTT	CCAAAC	TGGAAC	AACACT	CAACCC	TATCTC
6301	GGTCTA	TTCTTT		TGATTT	ATAAGG	GATTTT	GCCGAT	TTCGGC	CTATTG	GTTAAA	AAATGA
6361	GCTGAT	TTAACA		AAAATT	TAACGC	GAATTT	TAACAA	AATATT	AACGCT	TACAAT	TTAGGT
6421	GGCACT	TTTCGG		GGAAAT	GTGCGC	GGAACC	CCTATT	TGTTTA	TTTTTC	TAAATA	CATTCA
6481	AATATG	TATCCG		CTCATG	AGACAA	TAACCC	TGATAA	ATGCTT	CAATAA	TATTGA	AAAAGG
6541	AAGAGT	ATGAGT		ATTCAA	CATTTC	CGTGTC	GCCCTT	ATTCCC	TTTTTT	GCGGCA	TTTTGC
6601	CTTCCT	GTTTTT		GCTCAC	CCAGAA	ACGCTG	GTGAAA	GTAAAA	GATGCT	GAAGAT	CAGTTG
6661	GGTGCA	CGAGTG		GGTTAC	ATCGAA	CTGGAT	CTCAAC	AGCGGT	AAGATC	CTTGAG	AGTTTT
6721	CGCCCC	GAAGAA		CGTTTT	CCAATG	ATGAGC	ACTTTT	AAAGTT	CTGCTA	TGTGGC	GCGGTA
6781	TTATCC	CGTATT		GACGCC	GGGCAA	GAGCAA	CTCGGT	CGCCGC	ATACAC	TATTCT	CAGAAT
6841	GACTTG	GTTGAG		TACTCA	CCAGTC	ACAGAA	AAGCAT	CTTACG	GATGGC	ATGACA	GTAAGA
6901	GAATTA	TGCAGT		GCTGCC	ATAACC	ATGAGT	GATAAC	ACTGCG	GCCAAC	TTACTT	CTGACA
6961	ACGATC	GGAGGA		CCGAAG	GAGCTA	ACCGCT	TTTTTG	CACAAC	ATGGGG	GATCAT	GTAACT
7021	CGCCTT	GATCGT		TGGGAA	CCGGAG	CTGAAT	GAAGCC	ATACCA	AACGAC	GAGCGT	GACACC
7081	ACGATG	CCTGTA		GCAATG	GCAACA	ACGTTG	CGCAAA	CTATTA	ACTGGC	GAACTA	CTTACT
7141	CTAGCT	TCCCGG		CAACAA	TTAATA	GACTGG	ATGGAG	GCGGAT	AAAGTT	GCAGGA	CCACTT
7201	CTGCGC	TCGGCC		CTTCCG	GCTGGC	TGGTTT	ATTGCT	GATAAA	TCTGGA	GCCGGT	GAGCGT
7261	GGGTCT	CGCGGT		ATCATT	GCAGCA	CTGGGG	CCAGAT	GGTAAG	CCCTCC	CGTATC	GTAGTT
7321	ATCTAC	ACGACG		GGGAGT	CAGGCA	ACTATG	GATGAA	CGAAAT	AGACAG	ATCGCT	GAGATA
7381	GGTGCC	TCACTG		ATTAAG	CATTGG	TAACTG	TCAGAC	CAAGTT	TACTCA	TATATA	CTTTAG
7441	ATTGAT	TTAAAA		CTTCAT	TTTTAA	TTTAAA	AGGATC	TAGGTG	AAGATC	CTTTTT	GATAAT
7501	CTCATG	ACCAAA		ATCCCT	TAACGT	GAGTTT	TCGTTC	CACTGA	GCGTCA	GACCCC	GTAGAA
7561	AAGATC	AAAGGA		TCTTCT	TGAGAT	CCTTTT	TTTCTG	CGCGTA	ATCTGC	TGCTTG	CAAACA
7621	AAAAAA	CCACCG		CTACCA	GCGGTG	GTTTGT	TTGCCG	GATCAA	GAGCTA	CCAACT	CTTTTT
7681	CCGAAG	GTAACT		GGCTTC	AGCAGA	GCGCAG	ATACCA	AATACT	GTTCTT	CTAGTG	TAGCCG
7741	TAGTTA	GGCCAC		CACTTC	AAGAAC	TCTGTA	GCACCG	CCTACA	TACCTC	GCTCTG	CTAATC
7801	CTGTTA	CCAGTG		GCTGCT	GCCAGT	GGCGAT	AAGTCG	TGTCTT	ACCGGG	TTGGAC	TCAAGA
7861	CGATAG	TTACCG		GATAAG	GCGCAG	CGGTCG	GGCTGA	ACGGGG	GGTTCG	TGCACA	CAGCCC
7921	AGCTTG	GAGCGA		ACGACC	TACACC	GAACTG	AGATAC	CTACAG	CGTGAG	CTATGA	GAAAGC
7981	GCCACG	CTTCCC		GAAGGG	AGAAAG	GCGGAC	AGGTAT	CCGGTA	AGCGGC	AGGGTC	GGAACA
8041	GGAGAG	CGCACG		AGGGAG	CTTCCA	GGGGGA	AACGCC	TGGTAT	CTTTAT	AGTCCT	GTCGGG
8101	TTTCGC	CACCTC		TGACTT	GAGCGT	CGATTT	TTGTGA	TGCTCG	TCAGGG	GGGCGG	AGCCTA
8161	TGGAAA	AACGCC		AGCAAC	GCGGCC	TTTTTA	CGGTTC	CTGGCC	TTTTGC	TGGCCT	TTTGCT
8221	CACATG	TTCTTT		CCTGCG	TTATCC	CCTGAT	TCTGTG	GATAAC	CGTATT	ACCGCC	TTTGAG
8281	TGAGCT	GATACC		GCTCGC	CGCAGC	CGAACG	ACCGAG	CGCAGC	GAGTCA	GTGAGC	GAGGAA
8341	GCGGAA	GAGCGC		CCAATA	CGCAAA	CCGCCT	CTCCCC	GCGCGT	TGGCCG	ATTCAT	TAATGC
8401	AGCTGG	CACGAC		AGGTTT	CCCGAC	TGGAAA	GCGGGC	AGTGAG	CGCAAC	GCAATT	AATGTG
8461	AGTTAG	CTCACT		CATTAG	GCACCC	CAGGCT	TTACAC	TTTATG	CTTCCG	GCTCGT	ATGTTG
8521	TGTGGA	ATTGTG		AGCGGA	TAACAA	TTTCAC	ACAGGA	AACAGC	TATGAC	CATGAT	TACGCC
8581	AAGCGC	GCAATT		AACCCT	CACTAA	AGGGAA	CAAAAG	CTGGAG	CTGCAA	GCTT	
